# Tinnitus, Oscillopsia, and Hyperventilation-Induced Nystagmus: Vestibular Paroxysmia

**Published:** 2016

**Authors:** Bryan K Ward, Daniel R Gold

**Affiliations:** 1 Department of Otolaryngology-Head and Neck Surgery, Johns Hopkins University; 2 Departments of Neurology, Johns Hopkins University; 3 Departments of Neurosurgery, Johns Hopkins University; 4 Departments of Ophthalmology, Johns Hopkins University

**Keywords:** vestibular, vestibular paroxysmia, vascular compression, oscillopsia, nystagmus

## Abstract

Vestibular paroxysmia is the name given to vascular compression of the vestibulocochlear nerve. Substantial evidence has been discovered in support of vascular compression of the trigeminal nerve as the etiology for trigeminal neuralgia, and effective therapies have been targeted to address this pathophysiology. Perhaps due to the common and often vaguely-described symptoms of dizziness and tinnitus, vascular compression of the vestibulocochlear nerve as a cause of symptoms has remained controversial. Recent clinical studies, however, have better defined diagnostic criteria for vestibular paroxysmia. In this report we discuss a case of vestibular paroxysmia, highlighting some findings of the condition that also uniquely separate it from other more common vestibular disorders. Finally, we discuss current clinical management of vestibular paroxysmia.

## Introduction/Background

Vestibular paroxysmia is the name given to the syndrome caused by vascular compression of the vestibulocochlear nerve. Chronic external pressure on a cranial nerve from an adjacent blood vessel is thought to lead to demyelination, decreasing its firing threshold and making the nerve susceptible to undesirable stimulation by a mechanism called ephaptic transmission [1]. From the Greek translation, ephaptic means ‘touching onto’, and this mechanism of neural transmission has become important for understanding synchronized central neural processes. Aberrant ephaptic transmission during vascular compression has gained wide acceptance as a cause of trigeminal neuralgia.

Vascular compression as a cause for vestibular symptoms was first suggested in the 1930s by neurosurgeon Walter Dandy, who proposed that this finding observed in some patients undergoing vestibular neurectomy may be an etiology for Menière’s disease [2]. In the 1960s neurosurgeon Peter Jannetta observed that in many patients with trigeminal neuralgia, loops of vasculature could be seen adjacent to the trigeminal nerve. He reasoned that pressure from the adjacent vasculature might cause symptoms and began surgically decompressing the nerves by placing small pads between the vessel and nerves [3]. Many patients experienced relief, and the surgery has since gained popularity with between 74% and 86% of patients experiencing immediate post-operative symptom *resolution* depending on the case series [4]. Jannetta suspected that similar compression syndromes might occur with other cranial nerves; compression syndromes involving the vestibulocochlear nerve (vestibular paroxysmia), facial nerve (hemifacial spasm), trochlear nerve (superior oblique myokymia), abducens andoculomotor nerves (ocular neuromyotonia) have since been recognized. We present a case of vestibular paroxysmia and discuss the management of this challenging and controversial clinical condition.

## Case Presentation

A 52 year-old woman awoke from sleep, bothered by a crackling noise in her left ear. She noticed vertical “waviness” to her vision, dizziness, and unsteadiness to her gait, tending to veer left. The symptoms lasted seconds to minutes, occurring up to hundreds of times daily with increasing frequency throughout the day. Whenever the crackling noise was experienced, she had unsteady gait and vertical jumping of vision. The attacks were spontaneous. Computed tomography (CT) imaging of the temporal bone was normal, without evidence of a labyrinthine dehiscence. Audiogram, electronystagmography and vestibular-evoked myogenic potentials were normal for the symptomatic ear. She experienced no benefit from prednisone, oral antibiotics or from a course of diuretics. She reported a history of motion sickness and had migraine headaches in the past, but not for over 25 years.

On exam, ocular motor examination was normal. Vestibular slow phases were normal for passive head rotation and for rapid head impulses in the plane of the horizontal semicircular canals. With video-oculography and removal of fixation, there was a left-beating nystagmus after hyperventilation. Dix-Hallpike maneuvers revealed the same left-beating component, regardless of tested direction. Auditory brainstem responses (ABR) showed delayed latency peaks and increased inter-peak latencies in the left ear and normal responses in the right. Magnetic resonance imaging (MRI) of the brain and internal auditory canal was performed

## Discussion/Conclusion

The above case is an example of vestibular paroxysmia. Vestibular paroxysmia is currently a diagnosis of exclusion, but common features include: brief attacks of vertigo or oscillopsia *(i.e.* the false sense that the visual surround is oscillating [5], presumably due to spontaneous nystagmus from transient vestibulocochlear nerve irritation) lasting seconds to minutes, with associated tinnitus, hearing changes or gait disturbance during attacks, measurable auditory or vestibular deficits, and efficacy of anti-epileptics [6,7]. Many patients develop nystagmus with hyperventilation [7], thought to be due to transient changes in conductivity across the demyelinated portion of the nerve during hyperventilation, causing excitatory or inhibitory patterns of nystagmus. Consequently, the symptoms of vestibular paroxysmia can be exercise-induced. Head movements or different head positions can trigger symptoms in some patients, presumably by increased neurovascular contact during certain positions. Finally, these patients may have prolonged wave latency on ABR [8], as was identified in this case, perhaps due to demyelination.

Prior to attributing a patient’s symptoms to vestibular paroxysmia, however, clinicians must exclude common conditions likebenign paroxysmal positional vertigo (BPPV), Menière’s disease, vestibular neuritis and vestibular migraine. This is usually possible with a thorough history and bedside vestibular/ocular motor examination. In the described case, symptoms were not exclusively triggered by head movements, and Dix-Hallpike and supine roll testing were negative making BPPV unlikely; audiometry and short-lived attacks were a typical of Menière’s disease; and the patient lacked symptoms consistent with vestibular migraine (although this diagnosis should be kept in mind in any case of unexplained dizziness/vertigo with normal audio vestibular testing and history of migraine and/or motion sensitivity). Furthermore, simple partial seizures can occasionally present with a tinnitus aura [8], however, we did not feel the frequency or duration of attacks or their association with vestibular symptoms were consistent with this diagnosis.

Despite sound scientific basis, vestibular paroxysmia remains controversial, due to both unclear diagnostic criteria, and the finding that many asymptomatic individuals can also have a vessel in proximity to a cranial nerve on MRI. Since the vast majority of patients with vestibular paroxysmia respond to carbamazepine or oxcarbazepine, a trial of one of these medications can be both diagnostic and therapeutic in cases where MRI demonstrated vascular compression is causal. The patient above was treated with carbamazepine, with long-term symptom relief. Some patients that do not respond eventually undergo surgical decompression of the vestibulocochlear nerve. While surgery has been effective in some cases [9,10], additional studies are needed to demonstrate the efficacy of this intervention.

## Figures and Tables

**Figure 1:**
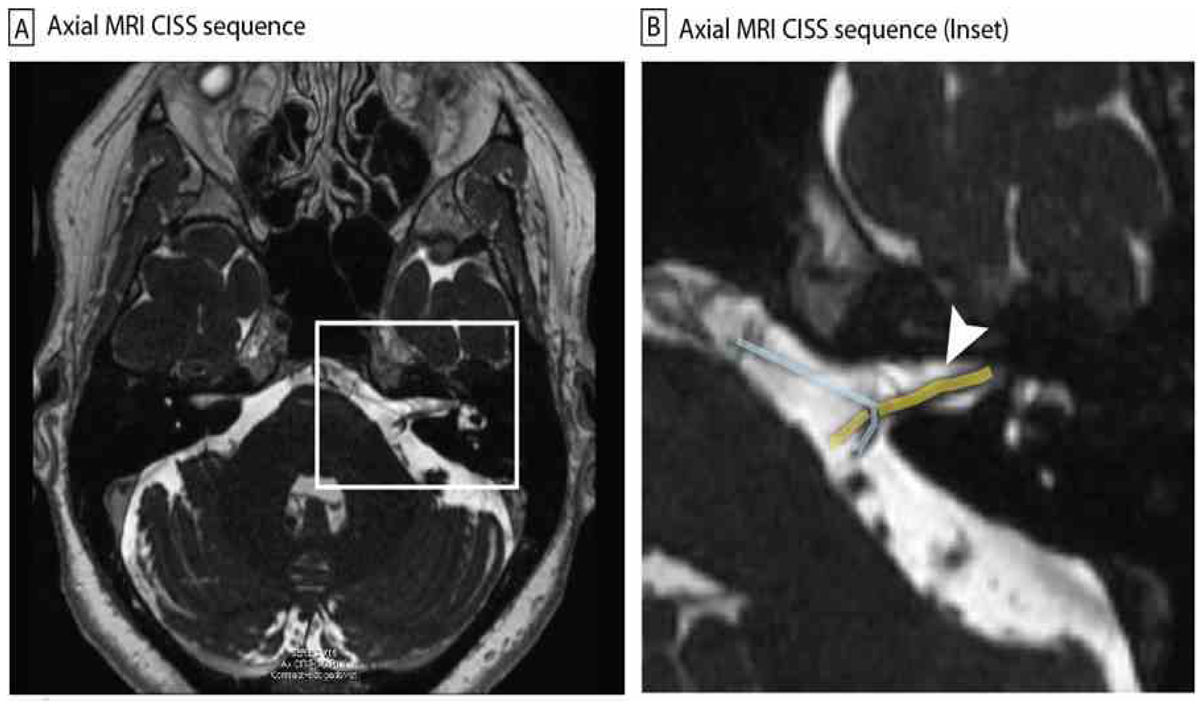
Axial constructive interference in steady state (CISS) sequence MRI imaging showing vascular compression at the internal auditory canal (IAC) (A). Inset (B) is magnified view of the left IAC (white arrow), with the nerves (yellow) and vessel (blue) highlighted for emphasis.

## Abbreviations

CT: Computed Tomography
ABR: Auditory Brainstem Responses
MRI: Magnetic Resonance Imaging
CISS: Constructive Interference in Steady State
IAC: Internal Auditory Canal
BPPV: Benign Paroxysmal Positional Vertigo
